# Supplementary material for: Imaging radiation dose in breast radiotherapy by X-ray CT calibration of Cherenkov light
Source: Nat Commun. 2020 May 8;11:2298. doi: 10.1038/s41467-020-16031-z (PMC7210272; doi:10.1038/s41467-020-16031-z)
Supplement: Supplementary file 1 — Supplementary Information [file 41467_2020_16031_MOESM1_ESM.pdf]

Imaging Radiation Dose in Breast Radiotherapy by X-ray CT Calibration of Cherenkov Light. (Supplementary Information)

Hachadorian et al.

Supplementary Note 1

The surface dose images were created in C-Dose software using the DICOM treatment plan (Supplementary Figure 1(a)) exported from Eclipse (Varian), determined by taking a weighted average of the sub-surface dose volume. This average was performed along the vectors normal to the patient's skin surface, sampled, then projected onto the surface of the CT contour. The averaging was exponentially weighted to the 1/*e* depth of 5 mm (Supplementary Figure 1(b)). This average dose map was rendered and displayed in the C-Dose analysis software onto a 3D-rendered version of the patient CT scan. The perspective of the rendered image matched the perspective of the Cherenkov camera in the treatment room, enabling a direct comparison of the planned surface dose image against acquired Cherenkov image from the viewpoint of the Cherenkov camera. To dose-normalize each Cherenkov image, these images were exported without graphic shadowing or the patient CT contour enabled, and co-registered using fiducial point selection. Dose normalization allows the user to directly observe the amount of Cherenkov radiation emitted per unit dose deposited in a given patient.

Supplementary Note 2

To quantify mean HU values, a contour was created using the Eclipse treatment planning software (Varian) by extracting a 10 mm wall inward from the body structure in the regions below the skin surface. It has been shown that most optical Cherenkov light is attenuated by the *in vivo*-like characteristics of the human body (including interstitial blood, melanin etc.) within the first centimeter of tissue, thus the region of focus is 10 mm from the surface of the skin. An additional structure was the created using the 50% isodose line (Supplementary Figure 1(a), dose color-washed volume) to define the

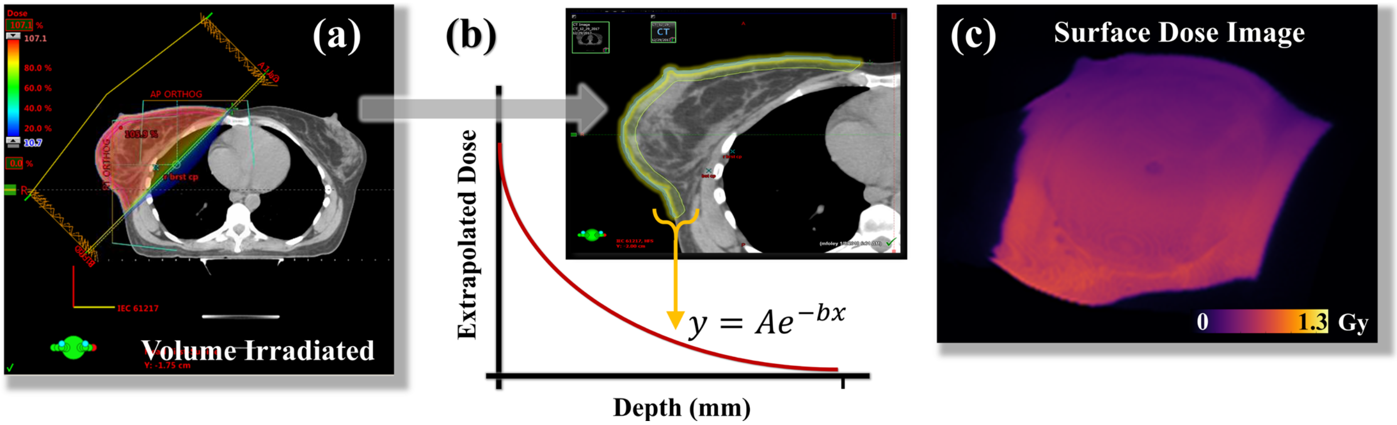

Supplementary Figure 1: Surface Dose Image Rendering. (a) The volume of breast irradiated is shown from an axial view in the treatment planning software (Eclipse). It is known that the optical Cherenkov emission being imaged comes primarily from well within the first 10 mm of tissue (more than 80% coming from the first 5 mm). The dose at the surface is weighted in the same manner that optical photons contribute to detected signal (b), which follows an exponential trend (Beer's law). When the extrapolated dose is projected onto the surface of the patient CT scan, the resulting image is shown in (c).

limits of the new wall structure. When only the intersecting regions of these structures are isolated, the result is a volume containing only first 10 mm of treated breast.

It is this average HU value that is plotted against dose-normalized Cherenkov intensity in Supplementary Figure 2. In the main article Figure 4, both 6 MV energy beams (RPO and LAO) are averaged for simplicity, because no stark differences in slope were discovered. When left separate (Supplementary Figure 2(a,d)), three of four linear correlations are reported to be significant. The insignificant trend ($p = 0.146$) corresponding to RPO 10X beams, is due to an increased average

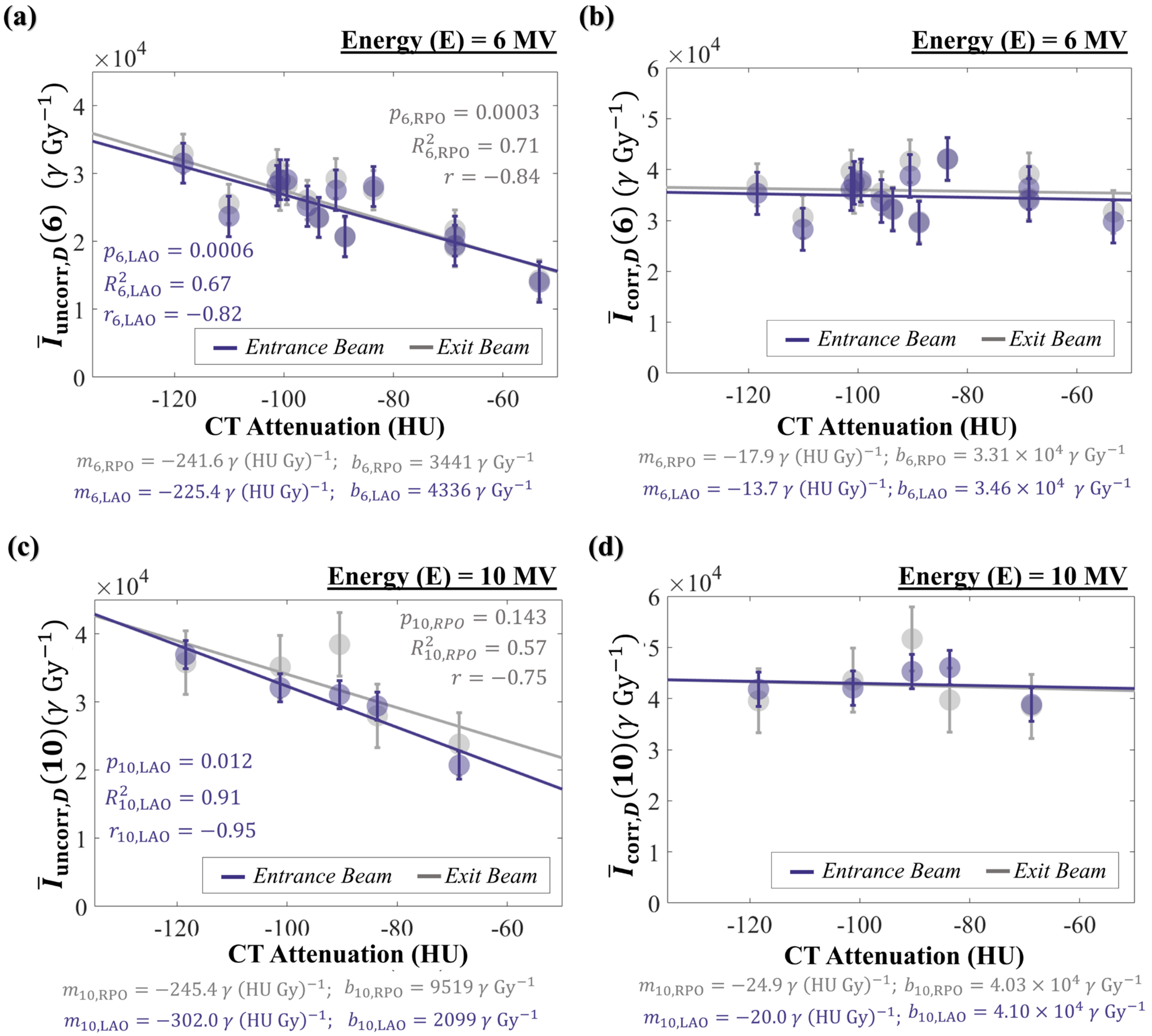

Supplementary Figure 2: Cherenkov (Dose-Normalized) relationship with CT radiodensity (HU). Relationships mapped between median Cherenkov light intensity (averaged over all fractions (mean)) and CT attenuation (HU) are reintroduced in (a), *n*=13 and (c), *n*=5, here, as they were in the main article Figure 4(c, d). To show the equalization of the data, used to compute the correction factors, the corrected plots are shown in (b) and (d). It can be observed that the average data from one patient in (c), Pt 7, is notably high, having to do with a substantial increase in signal around a deep skin fold in the upper arm. (Entrance/RPO $R^2 = 0.71$, $p = 0.0003$; Exit/LAO $R^2 = 0.67$, $p=0.0006$), where subfigure (d) does the same for the three patients receiving an additional pair of opposing 10 MV beams (Entrance/RPO $R^2 = 0.57$, $p = 0.143$; Exit/LAO $R^2 = 0.91$, $p=0.012$). Error bars shown represent the root mean square error. Pearson's correlation coefficient and *p*-value specifics can be found below in statistics and reproducibility following Supplementary Note 5.

intensity surrounding the deep upper arm fold of Pt 7. We suspect this can be largely contributed with increased signal around regions with skin folds due to a self-bolusing effect[1].

Supplementary Note 3

As can be observed in Figure 6, a given singular region of dose sampled from the surface dose image often corresponds to a range of Cherenkov intensities (scatter points appear stacked). The authorship attributes a majority of this variability to skin changes throughout the course of treatment, namely, adverse erythema-related side effects. Skin reddening and desiccation substantially attenuate the recorded Cherenkov emission, which has been mapped, using Pt 2 as a case study (Supplementary Figure 3). In Supplementary Figure 3(a,b) the reduction in emitted light is observable for both entrance and exit beams, respectively, beginning from Day 1 to Day 22. In (c), the median Cherenkov intensities for the entrance and exit beams were evaluated and plotted with respect to the day of treatment recorded. An approximate 250 count decrease in recorded photon intensity was mapped for the entrance beam, and a 320 count decrease was recorded for the exit beam.

It is possible that intra-patient Cherenkov emission variability could be accounted for using a crude reflectance reading. To follow the remote nature of data acquisition in this study, these measurements could be taken using the surface-guidance

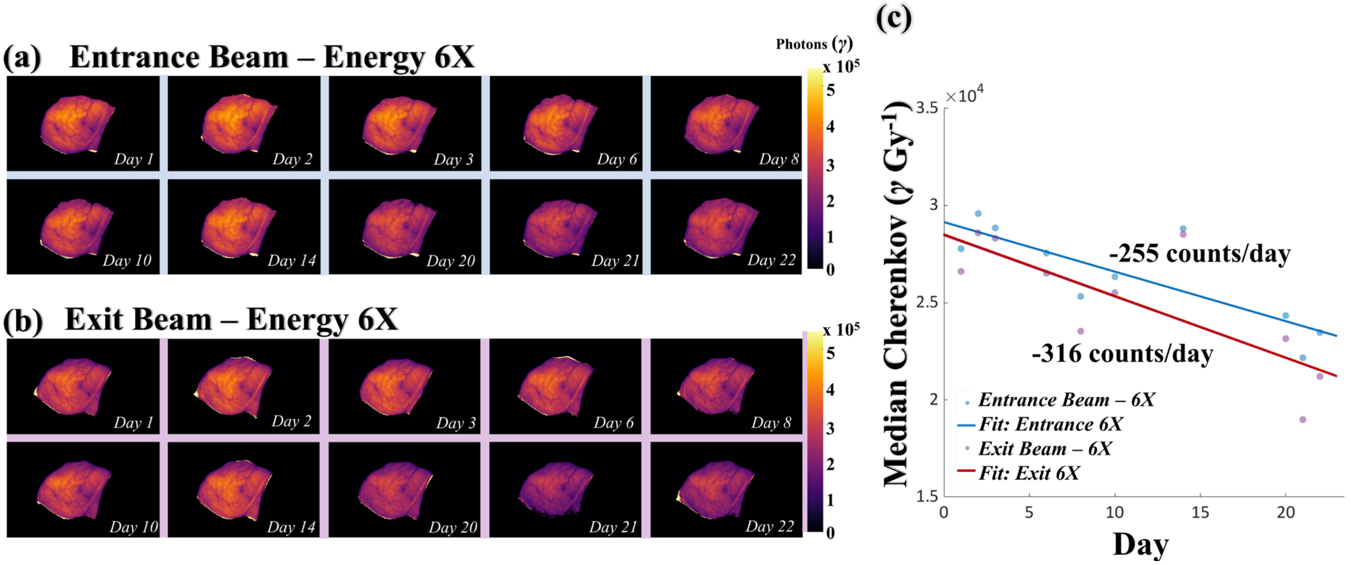

Supplementary Figure 3: Intra-fraction Changes. Ten recorded fractions from the treatment of Pt 2 are shown (dose-normalized) for the entrance beam (a) and exit beam (b). From day 1 to day 22, (*n*=10) a reduction in optical light emission from the tissue is observable. This reduction is attributed to skin reddening and desiccation, associated with erythema-related side effects. The median of each field was taken and plotted against the day of treatment. At roughly 250 and 320 count per day reductions in intensity, intra-patient changes that take place throughout the course of treatment contribute substantially to reduced linearity between Cherenkov light and dose (c). It is possible that the use of a cruse reflectance measurement using optical surface guidance systems as a light source could reduce this variability.

imaging systems in clinics, used to align the patient prior to treatment. This is one, proposed methodology currently underway, and will be evaluated in future work.

Supplementary Note 4

To verify the results seen in patient subjects, a controlled experiment was carried out to correlate the CT radiodensity (HU value) of underlying tissues with the Cherenkov output of those tissues. Phantoms were created from bovine tissues (80% raw muscle tissue and 20% adipose tissue) to mimic fibroglandular content with added adipose tallow (100%, bovine) to create phantoms of varying adipose concentrations (Supplementary Figure 4(a)). The resulting CT values ranged from -173

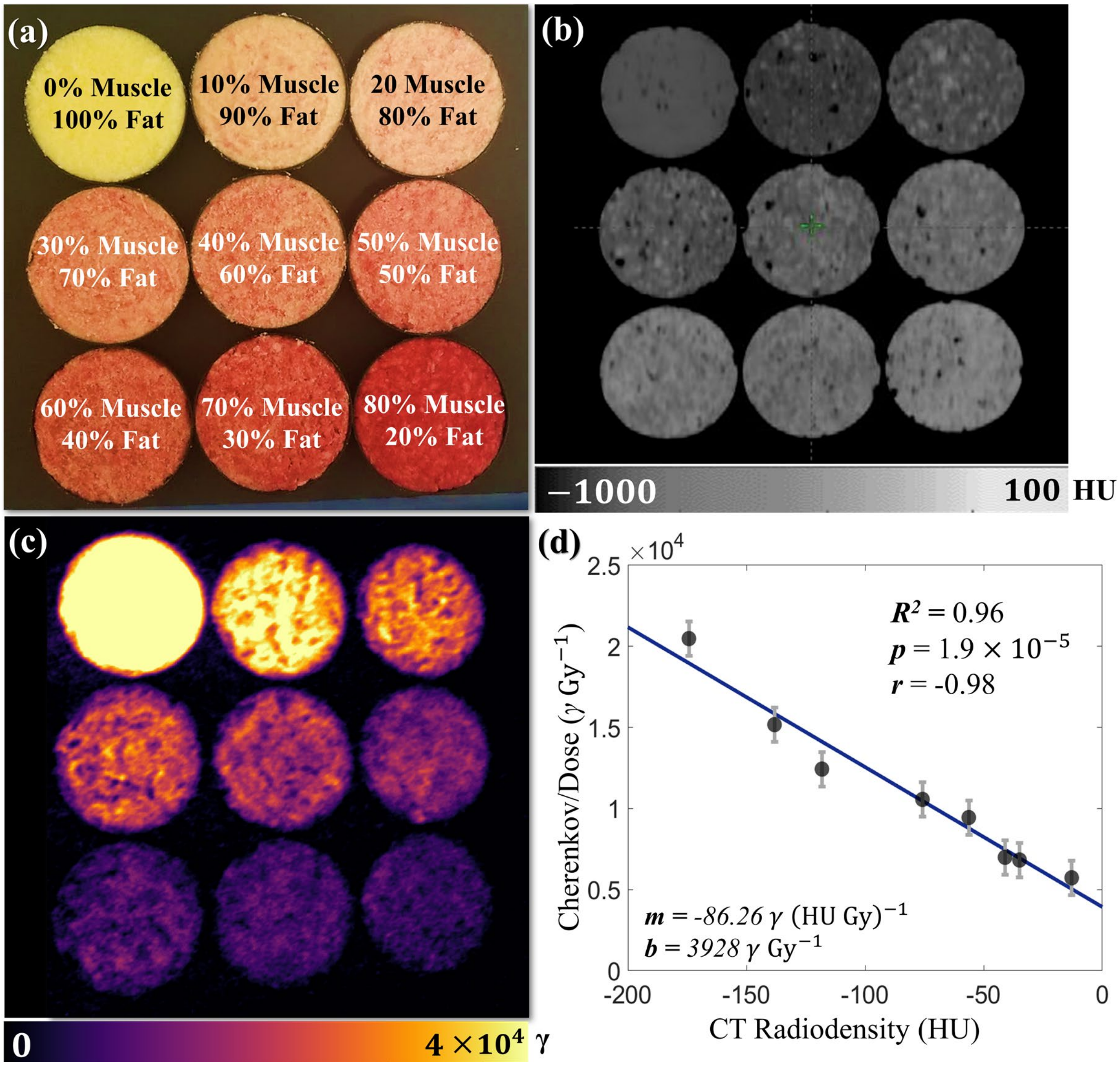

Supplementary Figure 4: Controlled HU/Cherenkov Calibration. In (a), the phantoms are laid out from most-fat containing to least fat-containing from left to right, top to bottom. In (b), the CT HU is shown as it appears in the treatment planning system with increasing radiodensity as the adipose content decreases. In (c), the Cherenkov image most certainly shows a reduced optical output from phantoms containing increased beef tissue content. In (d), n=8, the correlation between the CT radiodensity (HU) and the dose-normalized mean Cherenkov output is shown with error bars represented by the root mean square error. Pearson's correlation coefficient and p-value specifics can be found below in statistics and reproducibility following Supplementary Note 5.

HU to -12.9 HU, broadly encompassing breast tissue averages seen in our patient cohort. A 30 cm x 30 cm field beam was delivered to the phantoms in the configuration shown, where the treatment plan demonstrates even dosing of the phantoms (coefficient of variation σ/μ =1.9%). In (b), the CT scan of all nine phantom wells shows an increase in radiodensity with a decrease in percent adipose content. In (c) the reduction in output is visible with increased denser (fibroglandular-like) content. The correlation is mapped in (d), ($p = 1.9 \times 10^{-5}$, $r = -0.98$) with an $R^2 = 0.96$. Because 100% fat was not mixed the same way that the meat-containing samples did (which mixes in air), it was not consistent with the other samples and was not included in the calibration. Though in Supplementary Figure 4(c) it is clear that fat observably emits more Cherenkov light than does the more radiodense, meat-containing samples. To quantify the light emitted from each phantom and the surface dose in each phantom, circular ROI's were drawn in each image, mean values were taken, and the average Cherenkov intensity output in each phantom was divided by its respective average mean dose for dose normalization. As was observed in patients, the correlation was negative and linear. While this slope is notably less steep than what was observed in patients, we can confidently contribute this discrepancy to the many different factors which affect the tissue optical properties in patients, including primarily differences in skin. Nonetheless, the correlation in Supplementary Figure 4(d) demonstrates that CT number and Cherenkov light emission in radiotherapy is certainly one of these factors.

Supplementary Note 5

The patient treatment scar has the capacity to contribute substantial variability in the treatment field, based on the size of the patient surgical site. Large lumpectomy and partial mastectomy can lead to larger, deeper surgical scars than standard lumpectomy (for which the surgical scar contributes almost no notable effects to the Cherenkov whole-field median intensity). Ultimately, a more sophisticated, pixel-by-pixel type correction method could substantially aid in correcting for surgical scars by producing an image of surface weighted CT values, in contrast to the gross averaging of the most superficial 10 mm of treated tissues, which was done in this study. This would be carried through using a similar sampling as presented in Supplementary Figure 1: Surface Dose Image Rendering, but used to sample the patient CT scan instead of the patient treatment plan. The method for correction could remain the same, but the input would change from one scalar value to an image of CT surface pixels to accommodate spatial differences in the treated region.

To evaluate a worst-case scenario in the context of this study, Pt 10 (having the largest, deepest, most attenuating surgical scar) images were assessed quantitatively, where it was found between images including and excluding the surgical scar (Supplementary Figure 5), only a 2.3% increase was contributed to the Cherenkov median field intensity. As the technique becomes more refined, this will become a more important consideration. Though with large-scale tissue optical properties contributing to over 40% optical emission variability[2], these corrections are our first priority and primary concern for this study.

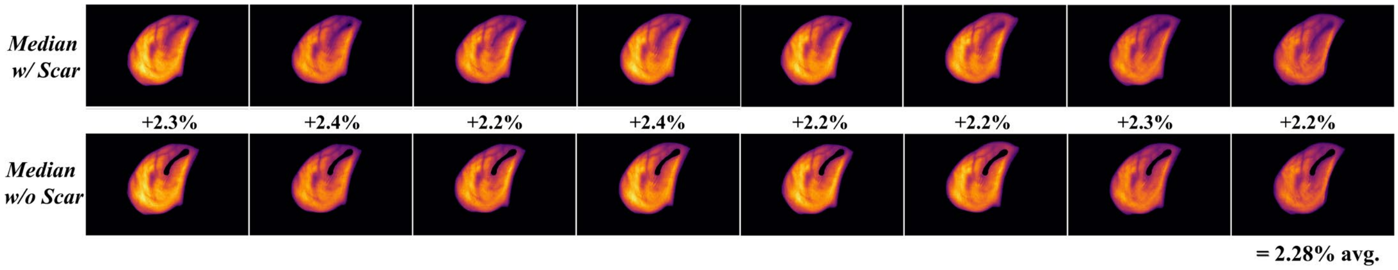

Supplementary Figure 5: Influence of the Surgical Scar on the Cherenkov Intensity. The surgical scar (Pt 10) was masked out to evaluate the Cherenkov intensity with and without the influence of the scar attenuation. Without, an average of 2.28% increase was observed.

Statistics and Reproduceability:

The Pearson's correlation coefficient and p-values were computed using MATLAB function corrcoef. As adapted from [3]: "The p-value is computed by transforming the correlation to create a t-statistic having $N - 2$ degrees of freedom, where N is the number of rows of X. The confidence bounds are based on an asymptotic normal distribution of $0.5 * \log((1 + r)/(1 - r))$, with an approximate variance equal to $1/(N - 3)$. These bounds are accurate for large samples when X has a multivariate normal distribution. The 'pairwise' option can produce an r matrix that is not positive definite… Default is 0.05 for 95% confidence intervals."

Supplementary References:

[1] Kole, A. J., Kole, L., & Moran, M. S. ,Acute radiation dermatitis in breast cancer patients: challenges and solutions. Breast Cancer: Targets and Therapy, 9, 313, (2017).

[2] Zhang, R., et al., Beam and tissue factors affecting Cherenkov image intensity for quantitative entrance and exit dosimetry on human tissue. J Biophotonics, 10(5): p. 645-656, (2017).

[3] MATHWORKS, Help Center: Correlation Coefficients "corrcoef" https://www.mathworks.com/help/finance/corrcoef.html, (2006).
