# Supplementary material for: Imaging radiation dose in breast radiotherapy by X-ray CT calibration of Cherenkov light
Source: Nat Commun. 2020 May 8;11:2298. doi: 10.1038/s41467-020-16031-z (PMC7210272; doi:10.1038/s41467-020-16031-z)
Supplement: Supplementary file 3 — Reporting Summary [file 41467_2020_16031_MOESM3_ESM.pdf]

nature research

Corresponding author(s): Lesley.A.Jarvis@hitchcock.org

Last updated by author(s): 03/24/2020

Reporting Summary

Nature Research wishes to improve the reproducibility of the work that we publish. This form provides structure for consistency and transparency in reporting. For further information on Nature Research policies, see Authors & Referees and the Editorial Policy Checklist.

Statistics

For all statistical analyses, confirm that the following items are present in the figure legend, table legend, main text, or Methods section.

n/a	Confirmed	
☐	☒	The exact sample size (*n*) for each experimental group/condition, given as a discrete number and unit of measurement
☐	☒	A statement on whether measurements were taken from distinct samples or whether the same sample was measured repeatedly
☐	☒	The statistical test(s) used AND whether they are one- or two-sided<br>*Only common tests should be described solely by name; describe more complex techniques in the Methods section.*
☐	☒	A description of all covariates tested
☐	☒	A description of any assumptions or corrections, such as tests of normality and adjustment for multiple comparisons
☐	☒	A full description of the statistical parameters including central tendency (e.g. means) or other basic estimates (e.g. regression coefficient) AND variation (e.g. standard deviation) or associated estimates of uncertainty (e.g. confidence intervals)
☐	☒	For null hypothesis testing, the test statistic (e.g. *F*, *t*, *r*) with confidence intervals, effect sizes, degrees of freedom and *P* value noted<br>*Give P values as exact values whenever suitable.*
☒	☐	For Bayesian analysis, information on the choice of priors and Markov chain Monte Carlo settings
☒	☐	For hierarchical and complex designs, identification of the appropriate level for tests and full reporting of outcomes
☐	☒	Estimates of effect sizes (e.g. Cohen's *d*, Pearson's *r*), indicating how they were calculated

Our web collection on statistics for biologists contains articles on many of the points above.

Software and code

Policy information about availability of computer code

Data collection	C-Dose software (commercial, Dose Optics) was used to collect Cherenkov data in clinic, and was spatially and temporally filtered in real time. Commercially Available. Run and tested on Windows 10, Lenovo ThinkPad P51. Ensure that your PC meets the minimum specs (16GB ram, 500GB SSD, i7 processor, NVIDIA GTX 1050 Ti or better, Windows 10 Pro 64-bit, USB3).<br><br>CT Scans were taken using a GE LightSpeed CT Scanner.
Data analysis	Further thresholding of Cherenkov images, averaging of relevant pixels and fitting of data was carried out in MATLAB (commercially available, MathWorks). Computation of p-values, R-squared values and pairwise, linear correlation coefficients (r) were also computed in MATLAB. Run and tested on Version: R2018A. Currently Operating on Windows 10. No required non-standard hardware.<br><br>We confirm that this work is original and has not been published elsewhere nor is it currently under consideration for publication elsewhere. MATLAB scripts used in all image processing have been uploaded to the following public data repository https://github.com/rachaelhach/NATCOMM_Cherenkov_HU, along with an instructional README file.<br><br>I-MAGE-J (FIJI). Public Domain Software by the NIH, Free Online Download. Run and Tested on Version 1.52a, operating on Windows 10. Analysis of average HU in relevant tissue regions was done in Varian ECLIPSE Treatment Planning Software.

For manuscripts utilizing custom algorithms or software that are central to the research but not yet described in published literature, software must be made available to editors/reviewers. We strongly encourage code deposition in a community repository (e.g. GitHub). See the Nature Research guidelines for submitting code & software for further information.

Data

Policy information about availability of data

All manuscripts must include a data availability statement. This statement should provide the following information, where applicable:

- Accession codes, unique identifiers, or web links for publicly available datasets
- A list of figures that have associated raw data
- A description of any restrictions on data availability

Data are available on request due to patient confidentiality or other restrictions.

Field-specific reporting

Please select the one below that is the best fit for your research. If you are not sure, read the appropriate sections before making your selection.

☒ Life sciences ☐ Behavioural & social sciences ☐ Ecological, evolutionary & environmental sciences

For a reference copy of the document with all sections, see nature.com/documents/nr-reporting-summary-flat.pdf

Life sciences study design

All studies must disclose on these points even when the disclosure is negative.

Sample size	Sample size includes 13 patients to correlate the median Cherenkov emission per unit dose and the average CT radiodensity (HU) of the tissue irradiated. These 13 patients were imaged between 2 and 17 times over the course of treatment, yielding a total of 110 data sets.
Data exclusions	No relevant data was excluded.
Replication	Patient treatment was verified each day of radiation therapy. CT radio-density analysis was confirmed through analysis ImageJ (Open Source, NIH Image processing software).
Randomization	Randomization was not relevant to this study. Participants were a cohort of the same group: adult women being treated for malignant neoplasm of the right breast (right breast cancer).
Blinding	Blinding was not relevant to this study. Patients were not allocated into experimental groups and treatments were planned on a patient-by-patient basis.

Reporting for specific materials, systems and methods

We require information from authors about some types of materials, experimental systems and methods used in many studies. Here, indicate whether each material, system or method listed is relevant to your study. If you are not sure if a list item applies to your research, read the appropriate section before selecting a response.

Materials & experimental systems

n/a	Involved in the study	
☒	☐	Antibodies
☒	☐	Eukaryotic cell lines
☒	☐	Palaeontology
☒	☐	Animals and other organisms
☐	☒	Human research participants
☒	☐	Clinical data

Methods

n/a	Involved in the study	
☒	☐	ChIP-seq
☒	☐	Flow cytometry
☒	☐	MRI-based neuroimaging

Human research participants

Policy information about studies involving human research participants

Population characteristics	Population demographics includes adult (aged 18 and over) women of the Lebanon, NH catchment area being treated for breast radiotherapy.
Recruitment	Recruitment was carried out by the patient's Radiation Oncologist at DHMC, Dr. Lesley Jarvis. These patients are otherwise sound of mind, understand the study and have agreed to imaging. No known recruitment biases exist, though the demographic of patients included in this study are limited inherently by the NH/VT catchment area.
Ethics oversight	1. D-HH Human Research Protection Program (IRB - Institutional Review Board)<br>2. NCCC Data, Safety Monitoring, and Accrual Committee (DSMAC). DSMAC reviews the study yearly.

Note that full information on the approval of the study protocol must also be provided in the manuscript.
